# Adaptation of risk prediction equations for cardiovascular outcomes among patients with type 2 diabetes in real-world settings: a cross-institutional study using common data model approach

**DOI:** 10.1186/s12933-024-02320-0

**Published:** 2024-07-10

**Authors:** Chun-Ting Yang, Kah Suan Chong, Chi-Chuan Wang, Huang-Tz Ou, Shihchen Kuo

**Affiliations:** 1 Institute of Clinical Pharmacy and Pharmaceutical Sciences, College of Medicine, National Cheng Kung University, Tainan, Taiwan; 2 Division of Pharmacoepidemiology and Pharmacoeconomics, Department of Medicine, Brigham and Women’s Hospital, Harvard Medical School, Boston, MA USA; 3 School of Pharmacy, College of Medicine, National Taiwan University, Taipei, Taiwan; 4 Graduate Institute of Clinical Pharmacy, College of Medicine, National Taiwan University, Taipei, Taiwan; 5 Department of Pharmacy, National Taiwan University Hospital, Taipei, Taiwan; 6 Department of Pharmacy, College of Medicine, National Cheng Kung University, Tainan, Taiwan; 7 Division of Metabolism, Endocrinology and Diabetes, Department of Internal Medicine, University of Michigan Medical School, Ann Arbor, Michigan, USA

**Keywords:** Prediction model, Diabetes, Cardiovascular, Recalibration

## Abstract

**Objective:**

To adapt risk prediction equations for myocardial infarction (MI), stroke, and heart failure (HF) among patients with type 2 diabetes in real-world settings using cross-institutional electronic health records (EHRs) in Taiwan.

**Methods:**

The EHRs from two medical centers, National Cheng Kung University Hospital (NCKUH; 11,740 patients) and National Taiwan University Hospital (NTUH; 20,313 patients), were analyzed using the common data model approach. Risk equations for MI, stroke, and HF from UKPDS-OM2, RECODe, and CHIME models were adapted for external validation and recalibration. External validation was assessed by (1) discrimination, evaluated by the area under the receiver operating characteristic curve (AUROC) and (2) calibration, evaluated by calibration slopes and intercepts and the Greenwood–Nam–D’Agostino (GND) test. Recalibration was conducted for unsatisfactory calibration (*p*-value of GND test < 0.05) by adjusting the baseline hazards of original equations to address variations in patients’ cardiovascular risks across institutions.

**Results:**

The CHIME risk equations had acceptable discrimination (AUROC: 0.71–0.79) and better calibration than that for UKPDS-OM2 and RECODe, although the calibration remained unsatisfactory. After recalibration, the calibration slopes/intercepts of the CHIME-MI, CHIME-stroke, and CHIME-HF risk equations were 0.9848/− 0.0008, 1.1003/− 0.0046, and 0.9436/0.0063 in the NCKUH population and 1.1060/− 0.0011, 0.8714/0.0030, and 1.0476/− 0.0016 in the NTUH population, respectively. All the recalibrated risk equations showed satisfactory calibration (*p*-values of GND tests ≥ 0.05).

**Conclusions:**

We provide valid risk prediction equations for MI, stroke, and HF outcomes in Taiwanese type 2 diabetes populations. A framework for adapting risk equations across institutions is also proposed.

**Supplementary Information:**

The online version contains supplementary material available at 10.1186/s12933-024-02320-0.

## Background

The prevention and management of cardiovascular complications are the primary treatment goals for patients with type 2 diabetes given the substantial health and economic burdens caused by cardiovascular disease (CVD)-related disability and mortality at both patient and population levels [1–3]. Identifying effective strategies for averting CVD among patients with type 2 diabetes is a critical focus in diabetes care. Risk equations for predicting CVD have emerged as efficient tools to inform clinical decision-making regarding type 2 diabetes [4–10], including the personalized estimation of CVD risks based on individual patient attributes, characterization of subpopulations at a high risk of developing CVD, and long-term health and economic outcome assessments of anti-diabetic interventions [11].

However, whether existing CVD risk prediction equations can precisely capture the cardiovascular risk profiles of real-world populations with type 2 diabetes is of concern. Specifically, the majority of risk equations were developed using relatively dated data (e.g., data collected before 2010) [4–10], while the clinical management for type 2 diabetes has advanced substantially in recent decades with the launch of novel glucose-lowering agents and the evolution of clinical guidelines on the management of patients’ CVD risks. Moreover, the existing risk equations were primarily established using data from very selective and homogeneous patient populations enrolled in clinical trials [4–9] or intervention programs [10, 12], and therefore the generalizability of these risk equations to diverse real-world patient populations is limited. Hence, an adaptation process, including external validation and recalibration, is warranted to ensure the validity of applying existing risk equations to real-world type 2 diabetes patients.

Compared with claims database, where clinical biomarkers are typically unavailable and disease-specific registries are not commonly established for diseases with sizeable populations (e.g., type 2 diabetes), electronic health records (EHRs), which contain detailed and structured patient information, are a more valuable real-world data source for the adaption of risk equations for type 2 diabetes. Furthermore, multiple-site or cross-institutional collaborations using EHRs could be considered to enhance the generalizability of adapted risk equations to diverse real-world patient populations.

Against this background, we adapted existing CVD risk prediction equations for real-world type 2 diabetes cohorts by utilizing the EHRs from two medical centers in Taiwan to provide valid tools that can be used to assess the risk of developing CVD and support clinical decision-making for type 2 diabetes patients. Given the restrictions imposed by data usage policies, imperative of safeguarding the privacy of personal records at each institution, and inconsistent data formats across institutions, this cross-institutional study applied the common data model (CDM) approach [13, 14] to ensure the privacy of patients’ data, consistency in data analysis, and transparency across study sites.

## Method

### Data sources

The EHRs from the National Cheng Kung University Hospital (NCKUH) 2014–2019 and National Taiwan University Hospital (NTUH) 2012–2017 in Taiwan were utilized in this study. The time periods used to identify the study populations, risk predictors, and cardiovascular outcomes from the NCKUH and NTUH EHRs are shown in Supplementary Fig. 1. The CDM approach was applied to facilitate cross-database analyses [13, 14]. Briefly, the NCKUH and NTUH databases were first transformed into a study-specific CDM with six dimensions of data elements: Demographic, Diagnosis, Prescriptions, Laboratory Result, Vital Signs, and Smoking Behavior [15]. All study analyses (i.e., Steps 2–5 in Supplementary Fig. 2) were then performed using the CDM datasets for NCKUH and NTUH separately using common analytic protocols and computer programming codes to assure the consistency of analysis algorithms across study sites. Respecting the privacy of patient-level data in each hospital, only aggregated estimates generated from individual hospital sites were available to the research coordinators (C.T.Y. and K.S.C.) for the integration of study results. The CDM procedures for NCKUH and NTUH are detailed elsewhere [15, 16]. This study was approved by the Research Ethics Committees of NCKUH (A-ER-108,097) and NTUH (201808029RSA).

### Identification of appropriate CVD risk equations for adaptation

As shown in Supplementary Fig. 2, this study began with a search of the literature that reported concurrent risk prediction equations for three major CVDs, namely myocardial infarction (MI), stroke, and heart failure (HF), as the primary focus in clinical management for patients with type 2 diabetes. Next, only the risk equations that explicitly documented the following items were considered for adaptation: (1) statistical models of the risk equations (e.g., Cox proportional hazard models, Weibull models), (2) intercepts of the risk equations, (3) coefficients for the explanatory variables/risk predictors, and (4) operational definitions of the explanatory and predicted outcome variables. Then, the United Kingdom Prospective Diabetes Study Outcomes Model 2 (UKPDS-OM2) [4], Risk Equations for Complications Of type 2 Diabetes (RECODe) [5], and Chinese Hong Kong Integrated Modeling and Evaluation (CHIME) [17] models were selected. The details of the risk predictors and risk equations are available in Supplementary Tables 1 and 2, respectively.

### Identification of patients with type 2 diabetes for CVD risk equation adaptation

Patients who had at least two diagnosis records of type 2 diabetes (Supplementary Table 3) within one year were identified. The date of the first type 2 diabetes diagnosis was defined as the index date. We only included patients with at least one HbA1c record before the index date since HbA1c is a major treatment indicator of glucose control for patients with type 2 diabetes and is typically included as an essential risk predictor in risk prediction equations of diabetes-related complications. Patients without continuous follow-up visits in the hospital system were excluded to minimize the impact of data discontinuity, a common issue caused by the loss of follow-up in study patients among EHR-based research. Continuous follow-up was defined as the existence of at least one record of outpatient department visits, hospitalizations, emergency room visits, physical examinations, laboratory tests, or drug refills annually after the index date.

Study subjects were randomly split into training and testing datasets with a 1:1 ratio. The randomization process was iterated until no differences were found in the baseline patient characteristics and CVD event rates between these two datasets, as examined using the standardized mean difference (SMD) and Poisson distribution, respectively.

### Measurements of risk predictors and cardiovascular outcomes

The risk predictors included demographic characteristics, physical examinations, laboratory data, medical histories, and medication use (detailed in Supplementary Table 1). The demographic characteristics of study patients were measured at the index date and other risk predictors were measured at the 1-year baseline period of or at the index date. Patients’ medical histories and medication use were identified using the International Classification of Diseases (ICD) diagnosis codes and the World Health Organization Anatomical Therapeutic Chemical (WHO ATC) classification system, respectively. The fully conditional specification method, a widely used technique of multiple imputation [18], was applied to handle the missing values of continuous variables, including physical examinations and laboratory data.

When the data of risk predictors were unavailable in the EHRs, some assumptions were made. First, since patients’ diabetes onsets could not be ascertained in EHRs (e.g., patients might have been diagnosed with type 2 diabetes before they came to the two medical centers in this study), we assumed a 5-year diabetes duration for study patients based on the literature, which shows an average diabetes duration of 5 to 8 years among the Taiwanese type 2 diabetes populations treated with glucose-lowering agents [19, 20]. Additionally, due to the lack of smoking history and hemoglobin data in the NTUH’s EHRs, the NTUH population was assumed to be non-smokers and have a hemoglobin level of 12.5 g/dL, which is aligned with the average hemoglobin level of the NCKUH population.

The three CVD outcomes of interest, namely MI, stroke, and HF, were defined using ICD disease codes from hospitalization or emergency room visit records. The operational definitions of risk predictors and cardiovascular outcomes are detailed in Supplementary Table 3.

### External validation and recalibration of existing CVD risk equations

The external validation (discrimination and calibration) of existing CVD risk equations for our study cohorts were performed using the training dataset. The discrimination was determined using the area under the receiver operating characteristic curve (AUROC), where a value of AUROC ≥ 0.7 indicated acceptable discrimination [21]. The calibration was evaluated using the slope and intercept of the regression line that displays the relationship between predicted risks (*x* axis in calibration plots) and observed risks (*y* axis in calibration plots) of each CVD outcome by decile of predicted risks. A slope of 1 and an intercept of 0 indicated ideal calibration. The Greenwood–Nam–D’Agostino (GND) test was applied to examine the concordance between predicted and observed CVD risks, where a *p*-value of the test of less than 0.05 indicated a significant difference between the predicted and observed values, thereby indicating unsatisfactory calibration [5, 22].

For each CVD outcome, if none of the risk equations performed ideally for the study cohorts, defined as acceptable discrimination (AUROC ≥ 0.7) and satisfactory calibration (*p*-value of GND test ≥ 0.05), recalibrations of the risk equations were performed. Specifically, based on the recalibration method proposed by Shao et al. [23], we recalibrated the risk equations by adjusting the baseline hazards of the original risk equations through the addition of multipliers that addressed the variations in patients’ risks of developing CVDs across different medical institutions (hereinafter referred to as institutional multipliers). Then, if the performance was still unsatisfactory, further recalibrations were conducted within each stratum of the predicted risks of developing CVDs that accounted for the heterogeneity of patients’ risk profiles (Supplementary Table 4, Eq. [4]). The recalibration process is detailed in Supplementary Table 4.

### Internal validation of recalibrated CVD risk equations using testing dataset

Following the recalibration procedures, the performance of the recalibrated risk equations was examined using the testing dataset through (1) a calibration plot to assess whether the calibration slope and intercept of the recalibrated risk equations improved (i.e., closer to 1 and 0, respectively) over the original risk equations, and (2) a GND test to determine whether a desirable calibration outcome was achieved (i.e., *p*-value of test ≥ 0.05, indicating concordance between predicted and observed risks) [5, 22].

All statistical analyses were performed using SAS 9.4, except for the calculation of predicted risks from the CHIME model (statistical codes released by Quan et al. in the GitHub repository https://github.com/quan-group/CHIME), which was performed using R 4.1.3.

## Results

A total of 11,740 and 20,313 patients with type 2 diabetes were identified from NCKUH (mean age: 63.3 years) and NTUH (64.8 years), respectively (Supplementary Fig. 3). More than 80% of the study patients used non-insulin glucose-lowering agents and approximately 15% used insulin. The prevalence of established CVDs in the study patients ranged from 2.4% (MI) to 24.1% (ischemic heart disease). Details of the baseline patient characteristics are given in Supplementary Tables 5 and 6.

As shown in Supplementary Tables 7, 2.38%, 6.80%, and 8.65% of the NCKUH population developed MI, stroke, and HF, respectively, over a mean follow-up of 4.5 years. Among the NTUH population, 1.52%, 4.18%, and 3.54% of the patients developed MI, stroke, and HF, respectively, over a mean follow-up of 4.3 years.

Table 1 presents the discrimination and calibration results for three existing CVD risk equations for our study cohorts. The AUROC values for the UKPDS-OM2, RECODe, and CHIME models are in the ranges of 0.5726–0.6498, 0.6579–0.8084, and 0.7078–0.7907, respectively, among our study cohorts. Overall, all the CVD risk equations from UKPDS-OM2 and the stroke risk equation from RECODe demonstrated unsatisfactory discrimination (AUROC < 0.7) for Taiwanese type 2 diabetes populations, and all the CVD risk equations from the CHIME model yielded acceptable discrimination (AUROC ≥ 0.7). Additionally, the calibration slopes of all CVD risk equations from the CHIME model were closer to 1 compared with those obtained from UKPDS-OM2 and RECODe (Supplementary Figs. 4–6). The CVD risk equations from the CHIME model were therefore chosen for further recalibration.

**Table 1:** Performance of CVD risk equations from UKPDS-OM2, RECODe, and CHIME models among type 2 diabetes populations of NCKUH and NTUH

	NCKUH			NTUH		
	UKPDS-OM2	RECODe	CHIME model	UKPDS-OM2	RECODe	CHIME model
Myocardial infarction						
Discrimination						
AUROC	0.6213	0.7722*	0.7659*	0.6194	0.7681*	0.7751*
Calibration						
Slope	0.0039	0.2326	0.4747^†^	0.0225	0.1785	0.3774^†^
Intercept	0.0037	− 0.0009	− 0.0013	0.0054	− 0.0012	− 0.0037
*p*-value of GND test	< 0.001	< 0.001	< 0.001	< 0.001	< 0.001	< 0.001
Stroke						
Discrimination						
AUROC	0.6498	0.6579	0.7078*	0.6496	0.7001*	0.7705*
Calibration						
Slope	0.2633	0.6401	0.7382^†^	0.2484	0.5582	0.7982^†^
Intercept	0.0062	0.0495	− 0.0160	0.0075	0.0280	− 0.0363
*p*-value of GND test	< 0.001	< 0.001	< 0.001	< 0.001	< 0.001	< 0.001
Heart failure						
Discrimination						
AUROC	0.6390	0.8084*	0.7907*	0.5726	0.8050*	0.7770*
Calibration						
Slope	0.4767	0.5931	0.7804^†^	0.1597	0.3147	0.3666^†^
Intercept	0.0126	0.0214	0.0039	0.0068	0.0039	− 0.0007
*p*-value of GND test	< 0.001	< 0.001	0.05	< 0.001	< 0.001	< 0.001

*CVD* cardiovascular disease,* UKPDS-OM2* UK Prospective Diabetes Study Outcomes Model 2,* RECODe* Risk Equations for Complications Of type 2 Diabetes,* CHIME* Chinese Hong Kong Integrated Modeling and Evaluation,* NCKUH* National Cheng Kung University,* NTUH* National Taiwan University,* AUROC* area under the receiver operating characteristics curve,* GND* Greenwood–Nam–D’Agostino

^*^Acceptable discriminations are defined as AUROC values higher than or equal to 0.7

^†^A calibration slope of 1 and an intercept of 0 indicated ideal calibration. The risk equations from the CHIME model had slopes closer to 1 compared with those from UKPDS-OM2 and RECODe

**Fig. 1:**
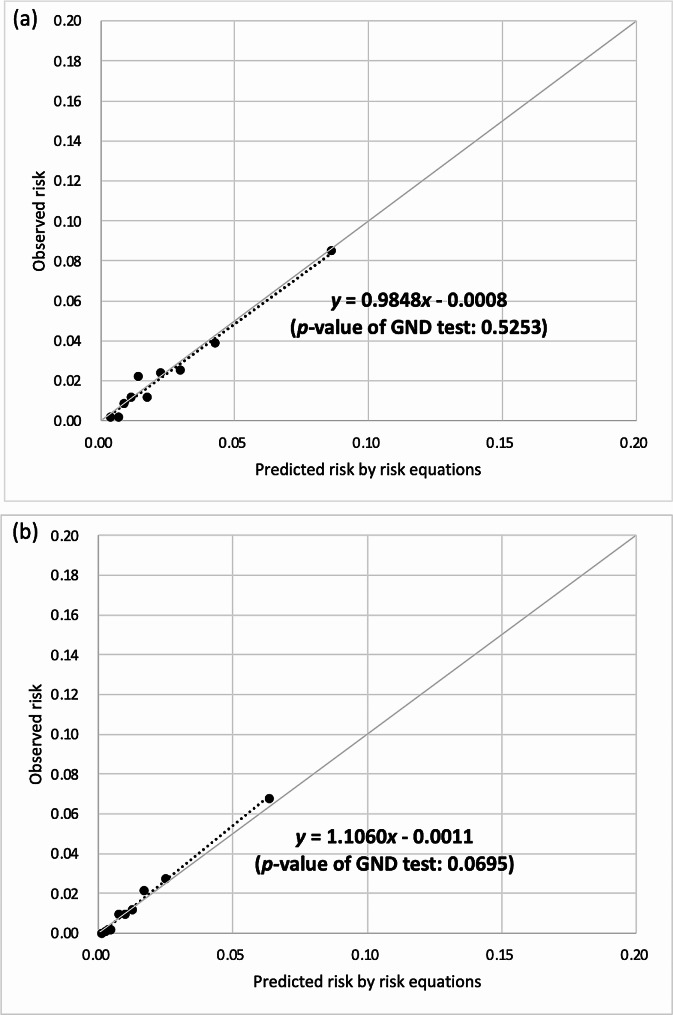
Recalibrated MI risk equations for type 2 diabetes populations of (**a**) NCKUH and (**b**) NTUH.* MI* myocardial infarction,* NCKUH* National Cheng Kung University Hospital,* NTUH* National Taiwan University Hospital,* GND* Greenwood–Nam–D’Agostino. The recalibrated risk equations of MI for (**a**) NCKUH and (**b**) NTUH populations are presented below, where Risk_recalibrated_ is the recalibrated risk estimate and Risk_CHIME_ is the risk estimate predicted using the original CHIME-MI risk equation: (**a**) Risk_recalibrated_ = exp [ln (Risk_CHIME_)– 0.7961947]. (**b**) Risk_recalibrated_ = exp [ln (Risk_CHIME_)– 1.55474 (if Risk_CHIME_ ≤ 2.5%)– 1.29655 (if 2.5% < Risk_CHIME_ < 10%)– 0.96124 (if Risk_CHIME_ ≥ 10%)]

**Fig. 2:**
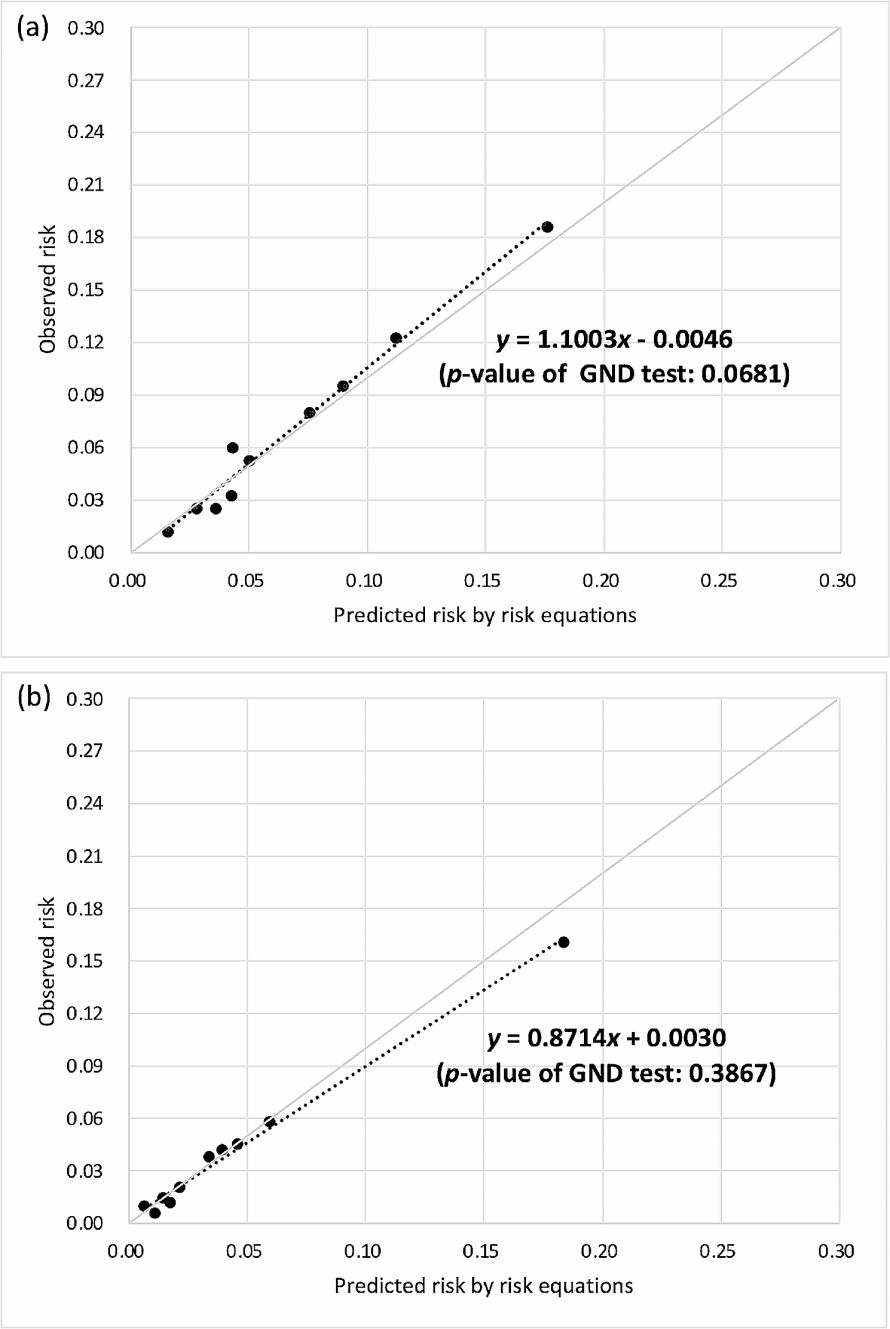
Recalibrated stroke risk equations for type 2 diabetes populations of (**a**) NCKUH and (**b**) NTUH. *NCKUH*, National Cheng Kung University Hospital; *NTUH*, National Taiwan University Hospital; GND, Greenwood-Nam-D’Agostino. The recalibrated risk equations of stroke for (**a**) NCKUH and (**b**) NTUH populations are presented below, where Risk_recalibrated_ is the recalibrated risk estimate and Risk_CHIME_ is the risk estimate predicted using the original CHIME-stroke risk equation: (**a**) Risk_recalibrated_= exp [ln (Risk_CHIME_)– 0.540353318 (if Risk_CHIME_ ≤ 8%)–0.726038047 (if 8% < Risk_CHIME_ < 11%)– 0.46372315 (if Risk_CHIME_≥ 11%)] (**b**) Risk_recalibrated_= exp [ln (Risk_CHIME_)– 1.37453272 (if Risk_CHIME_ ≤ 8.5%)– 1.00104983 (if 8.5% < Risk_CHIME_ < 17%)– 0.25721347 (if Risk_CHIME_≥ 17%)]

**Fig. 3:**
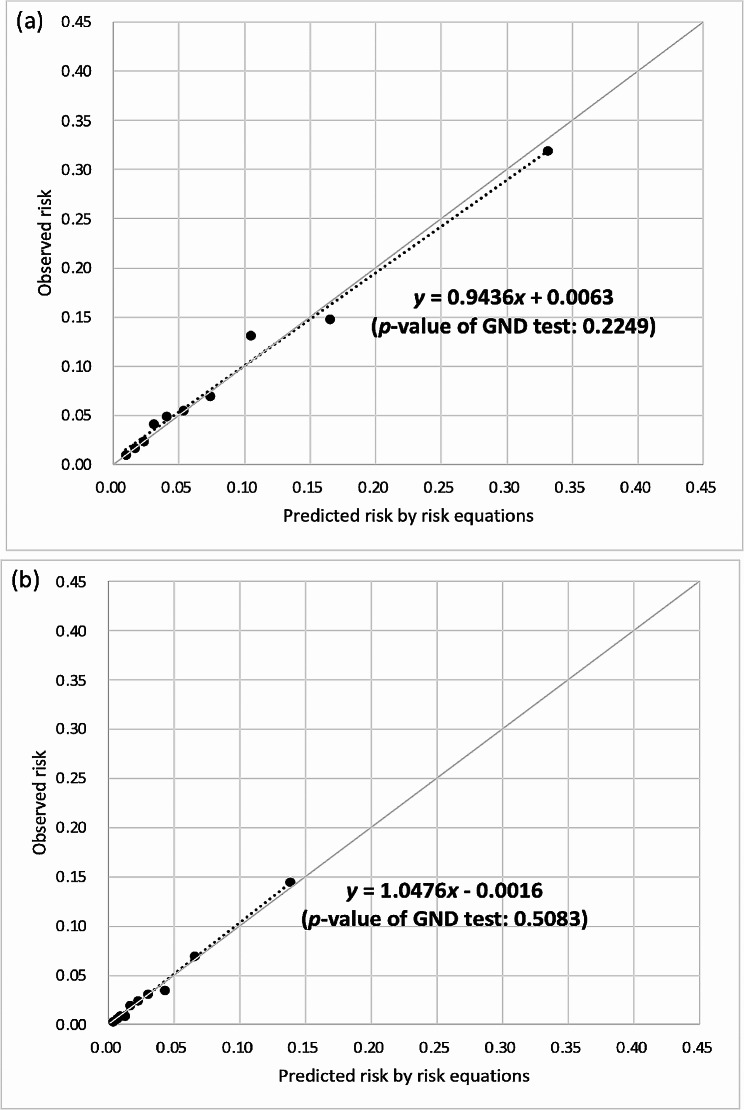
Recalibrated HF risk equations for type 2 diabetes populations of (**a**) NCKUH and (**b**) NTUH. *HF*, heart failure; *NCKUH*, National Cheng Kung University Hospital; *NTUH*, National Taiwan University Hospital; *GND*, Greenwood-Nam-D’Agostino. The recalibrated risk equations of HF for (**a**) NCKUH and (**b**) NTUH populations are presented below, where Risk_recalibrated_ is the recalibrated risk estimate and Risk_CHIME_ is the risk estimate predicted using the original CHIME-HF risk equation: (**a**) Risk_recalibrated_= exp [ln (Risk_CHIME_)– 0.201793786] (**b**) Risk_recalibrated_= exp [ln (Risk_CHIME_)– 1.022724239]

After recalibration (Figs. 1, 2 and 3), the calibration slopes/intercepts of the CHIME-MI, CHIME-stroke, and CHIME-HF risk equations improved from 0.4747/− 0.0013, 0.7382/− 0.0160, and 0.7804/0.0039 to 0.9848/− 0.0008, 1.1003/− 0.0046, and 0.9346/0.0063, respectively, among the NCKUH population. The calibration slopes/intercepts of the CHIME-MI, CHIME-stroke, and CHIME-HF risk equations improved from 0.3774/− 0.0037, 0.7982/− 0.0363, and 0.3666/− 0.0007 to 1.1060/− 0.0011, 0.8714/0.0030, and 1.0476/− 0.0016, respectively, among the NTUH population. All the recalibrated risk equations showed insignificant GND test results (*p*-values ≥ 0.05), suggesting satisfactory calibration.

## Discussion

This study provided valid risk equations for predicting the risks of MI, stroke, and HF in Taiwanese type 2 diabetes populations. Although the risk equations from the CHIME model were developed from real-world Asian type 2 diabetes populations, the calibration of these risk equations was unsatisfactory for Taiwanese type 2 diabetes populations. The substantially improved calibration of CVD risk equations from the CHIME model through our recalibration procedures suggest that the adaptation of risk prediction equations for a study-specific population is crucial to ensure the validity of predicted risk estimates. Additionally, the methodological efforts in this study provide a framework for adapting risk equations across different healthcare systems or databases for future local/regional and global collaborations.

### Differences in external validation of CVD risk equations from UKPDS-OM2, RECODe, and CHIME models among real-world type 2 diabetes populations in Taiwan

Among the original CVD risk equations from the UKPDS-OM2, RECODe, and CHIME models, those from UKPDS-OM2 had the poorest performance for Taiwanese type 2 diabetes populations in terms of unsatisfactory discrimination and calibration. The poor performance of the UKPDS-OM2 risk equations may be explained as follows. UKPDS-OM2 was developed using data from a clinical trial patient cohort with type 2 diabetes recruited between 1977 and 1991, where the disease management strategies for type 2 diabetes significantly differed from the contemporary clinical practice. For instance, in addition to newer glucose-lowering agents for glycemic control, the use of other pharmacological agents (e.g., statin, angiotensin-converting enzyme inhibitor/angiotensin receptor blocker) to prevent vascular complications is currently a crucial treatment strategy for type 2 diabetes populations; these agents were less emphasized when the UKPDS trial was conducted. As provided in Supplementary Table 8, the UKPDS trial participants had lower utilization rates of anti-hyperlipidemia and anti-hypertensive drugs compared with the patient cohorts that were used to develop the RECODe and CHIME models and our study populations of NCKUH and NTUH. Moreover, the prevalence of smoking behavior was considerably high among the UKPDS trial participants, of whom 31% and 35% were current or past smokers, respectively. Because of the evolution of clinical practice guidelines for type 2 diabetes, the risk equations established from outdated patient cohorts may not be applicable to real-world populations in modern clinical practice, which affects the accuracy of predicted risk estimates. Previous studies have reported a substantial overestimation of cardiovascular risks when applying the UKPDS-OM2 risk equations to contemporary study cohorts, for both Caucasian [24] and Asian populations [17].

The better performance of the CHIME model, compared with that of UKPDS-OM2 and RECODe, for Taiwanese populations is expected to be due to the similarity in the race/ethnicity and study settings between the patient cohort for the CHIME model development and our study patients. Specifically, the CHIME model was established using Chinese and East Asian populations, giving it better predictive performance for Taiwanese populations. Moreover, unlike the UKPDS-OM2 and RECODe models, which were derived from clinical trial populations, the CHIME model was developed using data from daily practice settings, which better reflected the patient characteristics and the patterns of clinical outcomes among real-world populations. Even so, further recalibration efforts may be needed to enhance the performance of the risk equations for study-specific populations.

### Application of adapted CVD risk equations to Taiwanese type 2 diabetes populations

Although risk equations for predicting CVDs among Taiwanese patients with type 2 diabetes have been previously developed, their applicability to current real-world practice is of concern. For example, Li et al. established a stroke risk scoring system using patients who were diagnosed with type 2 diabetes during 2001–2004 and enrolled in the National Diabetes Care Management Program in Taiwan. However, data of the patients in this program provided information from a relatively dated and highly selective study cohort, which may thereby limit the generalizability of the risk scoring system [10]. Another study conducted by Lin et al. developed a disease model that predicted the occurrence of diabetes-related complications through a data-driven approach, but the practical implementation of this model (e.g., the statistical formula and model) was not explicitly provided in detail, which has hindered its wide application [12]. Moreover, the generalizability of Lin et al.’s findings is of concern because the study was conducted using patients enrolled in a diabetes pay-for-performance program that provides an enhanced quality of diabetes care and has been demonstrated to lower the risks of diabetes-related complications compared with those of type 2 diabetes patients under routine care settings [25–27].

Compared with previous studies [10, 12], the present study utilized a contemporary cohort to better capture risk profiles of real-world type 2 diabetes populations in modern clinical practice. Additionally, the statistical models, coefficients of risk predictors, and intercepts of regression models of the adapted/recalibrated risk equations are explicitly reported to ensure transparency and facilitate further applications of our work (Figs. 1, 2 and 3, Supplementary Table 2). Further applications may include the estimation of patient-level CVD risks to formulate individualized treatment plans, identification of high-risk populations for whom an aggressive prevention strategy can be considered, and prioritization of health and economic benefits of anti-diabetic interventions to inform healthcare resource allocation. However, it should be acknowledged that the predicted CVD risks from this study might be generalizable only to those who share similar risk profiles, in terms of baseline patient characteristics and outcome risks, with our study populations even though our study cohorts represented real-world patient populations with diverse clinical characteristics. Therefore, future investigations on the generalizability and flexibility of our risk equations with healthcare systems with broader coverage regarding geographic area, institutional characteristics (e.g., accreditation levels), and patient attributes are encouraged.

### Proposed framework for adaption of risk prediction equations across healthcare systems

The present study proposed a framework for risk prediction equation adaptation that is straightforward to implement for multi-database collaborations, regardless of database type (e.g., EHRs, registry, claims data). This framework does not necessitate the pooling of patient-level data, thereby minimizing the concern of data privacy during cross-database collaborations. In addition, the incorporation of the CDM approach reduces the inconsistency in analytic procedures between different study sites and further enhances the precision of analysis results. Moreover, this framework allows the periodic refinement of the risk prediction equations through the updating of model institutional multipliers based on new data from other institutions, thereby ensuring the sustainability of the adapted risk prediction equations.

The recalibration method used in this study assumed that the strength of associations between risk predictors and predicted CVD outcomes (i.e., the coefficients of the risk predictors) are compatible across different study populations. This was done because in our case, the major risk factors for diabetes-related cardiovascular complications and the strength of associations between them, such as (1) the crucial demographic and clinical characteristics that are associated with the development of CVDs among patients with type 2 diabetes and (2) the magnitude of potential protective or harmful effects of risk predictors on CVDs, have been extensively explored and well recognized [28]. However, caution should be taken when applying this recalibration method to other disease areas where the epidemiological evidence is still controversial or lacking.

Some limitations should be acknowledged. First, due to the lack of hemoglobin and smoking data in the EHRs of NTUH, all of the NTUH patients were assumed to have a normal hemoglobin level of 12.5 g/dL and to be non-smokers, leading to a possible underestimation of CVD risks. However, this concern might be minimized because consistent calibration results of the original CVD risk equations were found when the assumption was modified (i.e., assuming that all of the NTUH patients are smokers). Second, similar to other EHRs or claims database research, diabetes duration is usually difficult to determine due to the lack of definite information about diabetes onset. We thus assumed that all study patients had a diabetes duration of 5 years, which might weaken the discrimination of risk equations and accuracy of predicted risk estimates. However, other risk predictors in the risk equations, such as the history of diabetes-related complications and use of glucose-lowering agents, that are related to diabetes duration for patients with type 2 diabetes are based on empiric data from study populations, which may have partially accounted for the effect of diabetes duration on the predicted risk estimates. Third, the information bias due to loss of follow-up, another common limitation inherent to studies using EHRs, may not have been fully eliminated in this study. Hence, several efforts were made to minimize this concern, including the exclusion of patients without continuous follow-up visits in the study hospitals and those without any HbA1c records at baseline. Fourth, there were several variables with a high rate of missing data (over 50%), including blood pressure levels (due to not being well-structured in the EMRs of study hospitals during study periods) and white blood cell counts (because of not being routinely collected in clinical practice). With this regard, the multiple imputation on these variables was made and study results were consistent before and after the data imputation. Future research with the complete data is warranted to corroborate our findings. Lastly, further research is warranted to determine the generalizability of our established risk equations to patient cohorts from different health care systems, with different demographic and clinical characteristics, and in longer follow-up periods (i.e., more than 5 years).

## Conclusions

This study provided valid risk equations for predicting the risks of MI, stroke, and HF among Taiwanese patients with type 2 diabetes in real-world settings. The adapted risk prediction equations can facilitate future health economics and outcomes research. Future research and collaborations with a broader coverage of healthcare systems across regions are encouraged to enhance the applicability of our proposed framework.

### Supplementary Information

Below is the link to the electronic supplementary material.

Supplementary Material 1

## Data Availability

No datasets were generated or analysed during the current study.

## Abbreviations

MI: Myocardial infarction
NCKUH: National Cheng Kung University Hospital
NTUH: National Taiwan University Hospital
GND: Greenwood–Nam–D’Agostino
