# Supplementary material for: Adaptation of risk prediction equations for cardiovascular outcomes among patients with type 2 diabetes in real-world settings: a cross-institutional study using common data model approach
Source: Cardiovasc Diabetol. 2024 Jul 10;23:244. doi: 10.1186/s12933-024-02320-0 (PMC11238483; doi:10.1186/s12933-024-02320-0)
Supplement: Supplementary file 1 — Supplementary Material 1 [file 12933_2024_2320_MOESM1_ESM.docx]

**List of all centers and participating investigators contributing to this analysis**

**National Cheng Kung University Hospital (NCKUH)**

Huang-Tz Ou (principal investigator, PI), Chun-Ting Yang, Kah-Suan Chong, Shihchen Kuo

**National Taiwan University Hospital (NTUH)**

Chi-Chuan Wang (PI)

List of Supplementary Tables, Supplementary Figures

Supplementary Table 1. Risk predictors of cardiovascular outcomes for risk equations from UKPDS-OM2, RECODe, and CHIME models

Supplementary Table 2. Detailed risk equations for MI, stroke, and HF from UKPDS-OM2, RECODe, and CHIME models

Supplementary Table 3. Operational definitions of disease diagnosis and medication use

Supplementary Table 4. Logic of recalibration method using recalibrations of risk equations from CHIME model as example

Supplementary Table 5. Demographic and clinical characteristics of patients with type 2 diabetes from NCKUH

Supplementary Table 6. Demographic and clinical characteristics of patients with type 2 diabetes from NTUH

Supplementary Table 7. Observed cardiovascular outcomes among patients with type 2 diabetes identified from electronic health records of NCKUH and NTUH

Supplementary Table 8. Patient characteristics of development cohorts for UKPDS-OM2, RECODe, and CHIME models and study populations in present study

Supplementary Figure 1. Identification of study populations, risk predictors, and cardiovascular outcomes from NCKUH and NTUH EHRs

Supplementary Figure 2. Study flow of risk equation adaptations for target study populations

Abbreviations: AUROC, area under the receiver operating characteristics curve; GND, Greenwood-Nam-D’Agostino.

Supplementary Figure 3. Study flow diagram in (a) National Cheng Kung University Hospital and (b) National Taiwan University Hospital

Supplementary Figure 4. Calibrations of MI risk equations from UKPDS-OM2, RECODe, and CHIME models for patients with type 2 diabetes from (a) NCKUH and (b) NTUH

Supplementary Figure 5. Calibrations of stroke risk equations from UKPDS-OM2, RECODe, and CHIME models for patients with type 2 diabetes from (a) NCKUH and (b) NTUH

Supplementary Figure 6. Calibrations of HF risk equations from UKPDS-OM2, RECODe, and CHIME models for patients with type 2 diabetes from (a) NCKUH and (b) NTUH

Supplementary Table 1. Risk predictors of cardiovascular outcomes for risk equations from UKPDS-OM2, RECODe, and CHIME models

| Predictors | UKPDS-OM2 | | | RECODe | | | CHIME model | | |
| --- | --- | --- | --- | --- | --- | --- | --- | --- | --- |
|  | **MI** | **Stroke** | **HF** | **MI** | **Stroke** | **HF** | **MI** | **Stroke** | **HF** |
| Demographic characteristics |  |  |  |  |  |  |  |  |  |
| Age | V | V | V | V | V | V | V | V | V |
| Sex | V | V |  | V | V | V | V | V | V |
| Duration of diabetes | V | V | V |  |  |  | V | V | V |
| Smoking status | V | V |  | V | V | V | V |  | V |
| Medical history |  |  |  |  |  |  |  |  |  |
| Composite of cardiovascular diseases |  |  |  | V | V | V |  |  |  |
| Atrial fibrillation |  | V | V |  |  |  |  | V | V |
| Stroke | V |  |  |  |  |  |  | V |  |
| Ischemic heart disease | V | V |  |  |  |  | V |  | V |
| Myocardial infarction |  |  |  |  |  |  | V |  | V |
| Heart failure | V |  |  |  |  |  | V |  |  |
| Peripheral vascular disease | V |  | V |  |  |  |  |  |  |
| Amputation | V | V | V |  |  |  |  |  |  |
| Ulcer |  |  | V |  |  |  |  |  |  |
| Medication use |  |  |  |  |  |  |  |  |  |
| Insulin |  |  |  |  |  |  |  |  | V |
| Glucose-lowering agents (except insulin) |  |  |  |  |  |  |  |  | V |
| Anti-hypertensive drugs |  |  |  | V | V | V |  |  | V |
| Anti-hyperlipidemia drugs |  |  |  | V | V | V |  |  |  |
| Anticoagulants |  |  |  | V | V | V |  |  |  |
| Physical examination |  |  |  |  |  |  |  |  |  |
| Body mass index |  |  | V |  |  |  | V | V | V |
| Systolic blood pressure | V | V |  | V | V | V | V | V | V |
| Diastolic blood pressure |  |  |  |  |  |  | V | V | V |
| Laboratory data |  |  |  |  |  |  |  |  |  |
| HbA1c | V | V |  | V | V | V | V | V | V |
| Total cholesterol |  |  |  | V | V | V |  |  |  |
| Triglycerides |  |  |  |  |  |  |  |  | V |
| High-density lipoprotein | V |  |  | V | V | V | V | V | V |
| Low-density lipoprotein | V | V | V |  |  |  |  |  |  |
| Serum creatinine |  |  |  | V | V | V |  |  |  |
| Estimated glomerular filtration rate | V | V | V |  |  |  | V |  | V |
| Urine albumin-to-creatinine ratio | V | V | V | V | V | V |  |  |  |
| White blood cell | V | V |  |  |  |  | V | V | V |
| Hemoglobin |  |  |  |  |  |  | V | V | V |

Abbreviations: UKPDS-OM2, UK Prospective Diabetes Study Outcomes Model 2; RECODe, Risk Equations for Complications Of type 2 Diabetes; CHIME, Chinese Hong Kong Integrated Modeling and Evaluation; MI, myocardial infarction; HF, heart failure.

Supplementary Table 2. Detailed risk equations for MI, stroke, and HF from UKPDS-OM2, RECODe, and CHIME models

| United Kingdom Prospective Diabetes Study Outcomes Model 2 (UKPDS-OM2) | |
| --- | --- |
| 1. The integrated hazard of clinical outcomes at time *t* is   $\boldsymbol{H}\left( \boldsymbol{t} \vert\boldsymbol{x}_{\boldsymbol{i}} \right)\boldsymbol{=}\exp\boldsymbol{(}\boldsymbol{\lambda}\boldsymbol{+}\boldsymbol{\beta}_{\boldsymbol{j}}\boldsymbol{x}_{\boldsymbol{j}}\boldsymbol{)}\boldsymbol{t}^{\boldsymbol{\rho}}$  where *β* is the coefficients, *x* is the values for each covariate for an individual patient, and *t* is the duration of diabetes.   1. Probability of clinical outcome occurring in the interval *t* to *t*+1 is calculated as   $\mathbf{1-}\exp\left\{ \boldsymbol{H}\left( \boldsymbol{t} \vert\boldsymbol{x}_{\boldsymbol{j}} \right)\boldsymbol{-H}\left( \boldsymbol{t+1} \vert\boldsymbol{x}_{\boldsymbol{j}} \right) \right\}$  In the present study, the study patients were assumed to have an average diabetes duration of 5 years. | |
| MI | Male populations   1. The integrated hazard of MI at time *t* = exp(-8.791 + 0.045*age [years] – 0.830 [if Afro-Caribbean] + 0.279 [if Indian] + 0.108*HbA1c [%] – 0.049*HDL [per 0.1 mg/dL]+ 0.023*LDL [per 0.1 mg/dL] + 0.203 [if micro- or macro-albuminuria] + 0.340 [if PVD] + 0.046*SBP [per 10 mmHg] + 0.277 [if smoker] + 0.026*WBC [per 10^9^/L] + 0.743 [if amputation history] + 0.814 [if HF history] + 0.846 [if IHD history] + 0.448 [if stroke history]) x t 2. Annual probability of MI occurrence for patients with a diabetes duration of 5 years = 1 – exp{H(t=5\|x_j_) – H(t=6\|x_j_)}   Female populations   1. The integrated hazard of MI at time *t* = exp(-8.708 + 0.041*age [years] – 1.684 [if Afro-Caribbean] –0.280 [if eGFR<60] + 0.078*HbA1c [%] + 0.035*LDL [per 0.1 mg/dL if LDL>3.5 mg/dL] + 0.277 [if micro- or macro-albuminuria] + 0.469 [if PVD] + 0.056*SBP [per 10 mmHg] + 0.344 [if smoker] + 0.070*WBC [per 10^9^/L] + 0.853 [if HF history] + 0.876 [if IHD history]) x t^1.376^ 2. Annual probability of MI occurrence for patients with a diabetes duration of 5 years = 1 – exp{H(t=5\|x_j_) – H(t=6\|x_j_)} |
| Stroke | 1. The integrated hazard of stroke at time *t* = exp(-13.053 + 0.066*age [years] – 0.420 [if female] + 1.476 [if AF] –0.190 [if eGFR<60] + 0.092*HbA1c [%] + 0.016*LDL [per 0.1 mg/dL] + 0.420 [if micro- or macro-albuminuria] + 0.170*SBP [per 10 mmHg] + 0.331 [if smoker] + 0.040*WBC [per 10^9^/L] + 1.090 [if amputation history] + 0.481 [if IHD history]) x t^1.466^ 2. Annual probability of stroke occurrence for patients with a diabetes duration of 5 years = 1 – exp{H(t=5\|x_j_) – H(t=6\|x_j_)} |
| HF | 1. The integrated hazard of HF at time *t* = exp(-12.332 + 0.068*age [years] + 1.562 [if AF] + 0.072*BMI [kg/m^2^] – 0.220 [if eGFR<60] + 0.012*LDL [per 0.1 mg/dL] + 0.771 [if micro- or macro-albuminuria] + 0.479 [if PVD] + 0.658 [if amputation history] + 0.654 [if ulcer history]) x t^1.514^ 2. Annual probability of HF occurrence for patients with a diabetes duration of 5 years = 1 – exp{H(t=5\|x_j_) – H(t=6\|x_j_)} |
| Risk Equations for Complications Of type 2 Diabetes (RECODe) | |
| The 10-year risk of clinical outcomes is calculated as  $\text{10-year risk (\%)}\boldsymbol{=1-\lambda^exp(}\sum\boldsymbol{(\beta x)}\mathbf{-}\text{mean}\mathbf{(}\sum\boldsymbol{(\beta x}\mathbf{)))}$  where *β* is the coefficients and *x* is the values for each covariate for an individual patient. *λ* values were 0.93, 0.98, and 0.96 for MI, stroke, and HF, respectively. | |
| MI | $\text{10-year MI risk (\%)}=1-0.93^exp$(0.04363*age [years] – 0.20660 [if female] – 0.11630 [if black] + 0.23580 [if current smoker] – 0.00514*SBP [mmHg] + 0.96180 [if CVD history] – 0.12480 [if anti-hypertensive drug use] + 0.04699 [if anti-hyperlipidemia drug use] + 0.54400 [if anticoagulant use] + 0.21350*HbA1c [%] + 0.00019*TC [mg/dL] – 0.01358*HDL [mg/dL] + 0.08027*Scr [mg/dL] + 0.00042*UACR [mg/g] – 2.92) |
| Stroke | $\text{10-year stroke risk (\%)}=1-0.98^exp$(0.02896*age [years] – 0.00326 [if female] + 0.27160 [if black] + 0.16650 [if current smoker] + 0.01659*SBP [mmHg] + 0.41380 [if CVD history] + 0.15980 [if anti-hypertensive drug use] – 0.18870 [if anti-hyperlipidemia drug use] – 0.13870 [if anticoagulant use] + 0.33650*HbA1c [%] + 0.00171*TC [mg/dL] – 0.00639*HDL [mg/dL] + 0.59550*Scr [mg/dL] + 0.00030*UACR [mg/g] – 6.96) |
| HF | $\text{10-year HF risk (\%)}=1-0.96^exp$(0.05268*age [years] + 0.25290 [if female] – 0.04969 [if black] + 0.29050 [if current smoker] + 0.00121*SBP [mmHg] + 1.00700 [if CVD history] + 0.63890 [if anti-hypertensive drug use] – 0.11750 [if anti-hyperlipidemia drug use] + 0.73650 [if anticoagulant use] + 0.20920*HbA1c [%] – 0.00136*TC [mg/dL] – 0.01758*HDL [mg/dL] + 0.82140*Scr [mg/dL] + 0.00041*UACR [mg/g] – 5.15) |
| Chinese Hong Kong Integrated Modeling and Evaluation (CHIME) model | |
| MI | The 5-year risk of MI is calculated as  $P\mathrm{rob}\left\{ T\geq t \right\}=1-\Phi\left( \frac{\log\left( t \right)-X_{\beta}}{\text{2.230191}} \right)$  where *t* = 5 and X_β_ =  12.26059 – 0.03579856*age – (5.736116*10^−6^)(age − 45.86995)^3^ + (1.084958*10^−5^)(age − 61.54141)^3^ – (5.11346*10^−6^)(age − 79.12115)^3^ − 0.03495308*duration of type 2 diabetes – (1.18837*10^−6^)(duration of type 2 diabetes − 0.04654346)^3^ + (2.396591*10^−6^)(duration of type 2 diabetes − 5.045859)^3^ – (1.208221×10^−6^)(duration of type 2 diabetes − 9.963039)^3^  − 0.2054818*HbA1c + 0.03414138*(HbA1c − 5.6)^3^ − 0.04046386*(HbA1c − 6.1)^3^ + 0.006322478*(HbA1c − 8.8)^3^  − 0.006227825*SBP – (2.217406*10^−6^)(SBP − 116)^3^ + (4.264241*10^−6^)(SBP − 134)^3^ – (2.046836*10^−6^)(SBP − 153.5)^3^  + 0.02343682*DBP – (4.21439*10^−5^)(DBP − 64.6)^3^ + (8.354787*10^−5^)(DBP − 76.85714)^3^ – (4.140397*10−5)(DBP − 89.33333)^3^  + 0.9748291*HDL − 0.7939204*(HDL − 0.91)^3^ + 1.328147*(HDL − 1.27)^3^ − 0.5342268*(HDL − 1.805)^3^  + 0.04451049*BMI − 0.0001299349*(BMI−20.38)^3^ + 0.0002327653(BMI − 24.92)^3^ − 0.0001028305*(BMI − 30.65667)^3^  − 0.2741083*WBC + 0.0056222*(WBC − 5.024157)^3^ − 0.00924382*(WBC − 7.2)^3^ + 0.00362162*(WBC − 10.57778)^3^  + 0.1479684*Hgb + 0.0009171825*(Hgb − 11.39235)^3^ − 0.00202552*(Hgb − 13.64)^3^ + 0.001108338*(Hgb − 15.5)^3^  + 0.5173726 [if female] + 0.2012889 [if ex-smoker] + 0.3544502 [if non-smoker] − 0.198558 [if 60≤eGFR<90] − 0.6139906 [if 45≤eGFR<60] − 0.9136492 [if 30≤eGFR<45] − 1.081039 [if 15≤eGFR<30] − 1.538418 [eGFR<15] − 0.6108059 [if IHD history] − 0.4667935 [if HF history] − 0.5474388 [if MI history]. |
| Stroke | The 5-year risk of stroke is calculated as  $P\mathrm{rob}\left\{ T\geq t \right\}=1-\Phi\left( \frac{\log\left( t \right)-X_{\beta}}{\text{2.37858}} \right)$  where *t* = 5 and X_β_ =  15.82026 − 0.0704093*age + (1.485588*10^−5^)(age − 45.78782)3 – (2.823105*10−5)(age − 61.54141)^3^ + (1.337517*10^−5^)(age − 79.03901)^3^ − 0.1366587*duration of type 2 diabetes + 0.001241157*(duration of type 2 diabetes − 0.04654346)^3^ − 0.002503046*(duration of type 2 diabetes − 5.045859)^3^ + 0.001261889*(duration of type 2 diabetes − 9.963039)^3^  + 0.08792962*HbA1c − 0.03425862*(HbA1c − 5.6)^3^ + 0.04060281*(HbA1c − 6.1)^3^ − 0.006344189*(HbA1c − 8.8)^3^  − 0.01030808*SBP – (4.042218*10^−6^)(SBP − 116)^3^ + (7.805663*10^−6^)(SBP − 134)^3^ – (3.763445*10^−6^)(SBP − 153.3333)^3^  + 0.007178522*DBP – (3.620796*10^−5^)(DBP − 64.6)^3^ + (7.164348*10^−5^)(DBP − 76.83333)^3^ – (3.543552*10^−5^)(DBP−89.33333)^3^  + 0.6314516*HDL − 0.3696312*(HDL−0.91)^3^ + 0.6183549*(HDL − 1.27)^3^ − 0.2487238*(HDL − 1.805)^3^  + 0.04083765*BMI − 0.0002714759*(BMI − 20.39)^3^ + 0.0004861972*(BMI − 24.93)^3^ − 0.0002147213*(BMI − 30.67)^3^  − 0.2198664*WBC + 0.004713937*(WBC − 5.015)^3^ − 0.007767682*(WBC − 7.1975)^3^ + 0.003053745*(WBC − 10.56653)^3^  + 0.04818864*Hgb + 0.003859686*(Hgb − 11.4)^3^ − 0.008553899*(Hgb − 13.65)^3^ + 0.004694213*(Hgb − 15.5)^3^  + 0.3607541 [if female] − 0.3148034 [if diabetes] − 1.31932 [if cerebrovascular disease history] − 0.7094421 [if AF history]. |
| HF | The 5-year risk of HF is calculated as  $P\mathrm{rob}\left\{ T\geq t \right\}=\left[ 1+\exp\left( \frac{\log\left( t \right)-X_{\beta}}{\text{1.022004}} \right) \right]^{-1}$  where *t* = 5 and X_β_ =  9.953031 − 0.05613912*age – (1.04749*10^−5^)(age−45.62902)^3^ + (2.003714*10^−5^)(age − 61.37714)^3^ – (9.562235*10^−6^)(age − 78.62834)^3^ − 0.08761027*duration of type 2 diabetes + 0.0006354283*(duration of type 2 diabetes − 0.04654346)^3^ − 0.001259727*(duration of type 2 diabetes − 4.960986)^3^ + 0.0006242988*(duration of type 2 diabetes − 9.963039)^3^  + 0.02312769*HbA1c − 0.03805*(HbA1c − 5.6)^3^ + 0.04509629*(HbA1c−6.1)^3^ − 0.007046296*(HbA1c−8.8)^3^  + 0.008674065*SBP – (1.28893*10^−5^)(SBP − 116)^3^ + (2.478712*10^−5^)(SBP − 134)^3^ – (1.189782*10^−5^)(SBP − 153.5)^3^  + 0.02084512*DBP – (4.692768*10^−5^)(DBP − 64.75)^3^ + (9.291682*10^−5^)(DBP − 77)^3^ – (4.598913*10^−5^)(DBP−89.5)^3^  + 0.5125827*triglycerides − 0.1571353*(triglycerides − 0.73)^3^ + 0.230551*(triglycerides − 1.3)^3^ − 0.07341569*(triglycerides − 2.52)^3^  + 0.8481022*HDL − 0.8385584*(HDL − 0.91)^3^ + 1.40335*(HDL − 1.27)^3^ − 0.5647914*(HDL − 1.8045)^3^  + 0.01717963*BMI − 0.0009786591*(BMI − 20.39)^3^ + 0.001752005*(BMI − 24.91)^3^ − 0.000773346*(BMI − 30.63)^3^  − 0.2128226*WBC + 0.005080483*(WBC − 5.02)^3^ − 0.008350127*(WBC − 7.2)^3^ + 0.003269644*(WBC − 10.58736)^3^  + 0.2154893*Hgb − 0.002767901*(Hgb − 11.4)^3^ + 0.006190033*(Hgb − 13.66667)^3^ − 0.003422132*(Hgb−15.5)^3^  + 0.1770644 [if female] − 0.2958122 [if diabetes] + 0.01637165 [if ex-smoker] + 0.2246938 [if non-smoker]  − 0.3700754 [if 60≤eGFR<90] − 0.9045605 [if 45≤eGFR<60] − 1.129907 [if 30≤eGFR<45] − 1.404198 [if 15≤eGFR<30] − 1.503548 [eGFR<15] − 0.4162028 [if insulin use] − 0.1655605 [if use of non-insulin GLA] − 0.2025641 [if use of anti-hypertensive agents] − 0.5152853 [if IHD history] − 0.3621113 [if MI history] − 1.143309 [if AF history]. |

Abbreviations: MI, myocardial infarction; HF, heart failure; HDL, high-density lipoprotein; LDL, low-density lipoprotein; PVD, peripheral vascular disease; SBP, systolic blood pressure; WBC, white blood cell; HF, heart failure; IHD, ischemic heart disease; eGFR, estimated glomerular filtration rate; BMI, body mass index; CVD, cardiovascular disease; Scr, serum creatinine; UACR, urine albumin creatinine ratio; TC, total cholesterol; DBP, diastolic blood pressure; Hgb, hemoglobin; AF, atrial fibrillation.

Supplementary Table 3. Operational definitions of disease diagnosis and medication use

| Variables | Operational definitions | Data sources* |
| --- | --- | --- |
| Cohort identification (using ICD-9-CM and ICD-10-CM diagnosis codes) | | |
| T2D diagnosis | ICD-9: 250.x0 or 250.x2, x=0~9  ICD-10: E11 | OPD records |
| Medical history at baseline^†^ (using ICD-9-CM and ICD-10-CM diagnosis codes) | | |
| Composite CVD | Having any records of atrial fibrillation, stroke, ischemic heart disease, myocardial infarction, or heart failure | ER, IPD, and OPD records |
| Atrial fibrillation | ICD-9: 427.31, 427.32 / ICD-10: I48 |  |
| Stroke | ICD-9: 430, 431, 432, 433, 434, 436  ICD-10: I60, I61, I62, I63, I65, I66 |  |
| Ischemic heart disease | ICD 9: 410, 411, 412, 413, 414  ICD 10: I20, I21, I22, I23, I24, I25 |  |
| Myocardial infarction | ICD-9: 410, 412 / ICD-10: I21-I22, I252 |  |
| Heart failure | ICD-9: 428 / ICD-10: I50 |  |
| Peripheral vascular disease | ICD-9: 250.7, 442.3, 443.81, 443.9, 444.22, 785.4, 707.1  ICD-10: E08.5, E09.5, E10.5, E11.5, E13.5, I72.4, I73.9, I74.3, I70.23-I70.26, A48.0, L97, L98.4, E08.621, E08.622, E09.621, E09.622, E10.621, E10.622, E11.621, E11.622, E13.621, E13.622 |  |
| Amputation | ICD-9: 895, 896, 897  ICD-10: S48, S58, S68, S78, S88, S98 |  |
| Ulcer | ICD-9: 707.1 / ICD-10: L97, L984, E08.621, E08.622, E09.621, E09.622, E10.621, E10.622, E11.621, E11.622, E13.621, E13.622 |  |
| Medication use at baseline^a^ (using WHO ATC Classification System) | | |
| Insulin | A10A | OPD records |
| Non-insulin | A10B |  |
| Anti-hypertensive drugs | C02, C03, C04, C07, C08, C09 |  |
| Anti-hyperlipidemia drugs | C10 |  |
| Anti-thrombotic therapy |  |  |
| Cardiovascular outcomes (using ICD-9-CM and ICD-10-CM diagnosis codes) | | |
| Myocardial infarction | ICD-9: 410 / ICD-10: I21-I22 | ER and IPD records |
| Stroke | ICD-9: 430, 431, 432, 433, 434, 436  ICD-10: I60, I61, I62, I63, I65, I66 |  |
| Heart failure | ICD-9: 428 / ICD-10: I50 |  |

Abbreviations: ICD-9-CM, International Classification of Diseases, Ninth Revision, Clinical Modification; ICD-10-CM, International Classification of Diseases, Tenth Revision, Clinical Modification; OPD, outpatient department; CVD, cardiovascular disease; ER, emergency room; IPD, inpatient department; WHO, World Health Organization; ATC, Anatomical Therapeutic Chemical.

*Disease variables were identified as having any diagnosis records from data sources.

^†^Baseline characteristics were measured from a one-year period prior to or at the index date.

Supplementary Table 4. Logic of recalibration method using recalibrations of risk equations from CHIME model as example

| $\boldsymbol{hazard}_{\boldsymbol{it}}\mathbf{=}\boldsymbol{h}_{\boldsymbol{t}}\mathbf{*}\mathbf{e}^{\left( \boldsymbol{\beta}_{\boldsymbol{D.B.T.H}}\mathbf{*}\boldsymbol{D.B.T.H}_{\boldsymbol{it}}\mathbf{+}\boldsymbol{\gamma}_{\boldsymbol{health system}}\mathbf{*}\boldsymbol{health system}_{\boldsymbol{i}} \right)}$ (1) |
| --- |
| Equation (1) is the proposed function for calculating the event risk. $\text{hazard}_{\text{it}}$ denotes the event rate at time *t* for individual *i*. $\text{h}_{\text{t}}$ is the baseline hazard at time *t*. The vector of $\text{D.B.T.H}_{\text{it}}$ comprises the risk predictors of demographics, biomarkers, treatments, and histories of complications. The vector of $\text{β}_{\text{D.B.T.H}}$ comprises the coefficients for the corresponding risk factors in $\text{D.B.T.H}_{\text{it}}$. $\text{health system}_{\text{i}}$represents the health system where an individual *i* is covered. $\text{γ}_{\text{health system}}$represents the multiplier for each health system. |
| $\boldsymbol{hazard}_{\boldsymbol{it}}\mathbf{=}\boldsymbol{h}_{\boldsymbol{t}}\mathbf{*}\mathbf{e}^{\left( \boldsymbol{\beta}_{\boldsymbol{D.B.T.H}}\mathbf{*}\boldsymbol{D.B.T.H}_{\boldsymbol{it}}\mathbf{+}\boldsymbol{\gamma}_{\boldsymbol{CMS}}\mathbf{*}\boldsymbol{CMS}_{\boldsymbol{i}}\mathbf{+}\boldsymbol{\gamma}_{\boldsymbol{NCKUH}}\mathbf{*}\boldsymbol{NCKUH}_{\boldsymbol{i}} \mathbf{+}\boldsymbol{\gamma}_{\boldsymbol{NTUH}}\mathbf{*}\boldsymbol{NTUH}_{\boldsymbol{i}} \right)}$ $\mathbf{=}\boldsymbol{h}_{\boldsymbol{t}}\mathbf{*}\mathbf{e}^{\mathbf{(}\boldsymbol{\gamma}_{\boldsymbol{CMS}}\mathbf{+}\boldsymbol{\beta}_{\boldsymbol{D.B.T.H}}\mathbf{*}\boldsymbol{D.B.T.H}_{\boldsymbol{it}}\mathbf{)}}\mathbf{*}\mathbf{e}^{\mathbf{(}\boldsymbol{\gamma}_{\boldsymbol{CMS}}\mathbf{*}\boldsymbol{CMS}_{\boldsymbol{i}}\mathbf{+}\boldsymbol{\gamma}_{\boldsymbol{NCKUH}}\mathbf{*}\boldsymbol{NCKUH}_{\boldsymbol{i}}\mathbf{+}\boldsymbol{\gamma}_{\boldsymbol{NTUH}}\mathbf{*}\boldsymbol{NTUH}_{\boldsymbol{i}}\mathbf{-}\boldsymbol{\gamma}_{\boldsymbol{CMS}}\mathbf{)}}$ (2) |
| In Equation (2), an extension of Equation (1), $\text{γ}_{\text{CMS}}$, $\text{γ}_{\text{NCKUH}}$, and $\text{γ}_{\text{NTUH}}$ represent the multipliers for the Hong Kong Hospital Authority system, National Cheng Kung University Hospital, and National Taiwan University Hospital, respectively. $\text{h}_{\text{t}}\text{*}\text{e}^{\text{(}\text{γ}_{\text{CMS}}\text{)}}$is the baseline hazard of the CHIME model. $\text{h}_{\text{t}}\text{*}\text{e}^{\text{(}\text{γ}_{\text{CMS}}\text{ }\text{+}\text{ }\text{β}_{\text{D.B.T.H }}\text{* }\text{D.B.T.H}_{\text{it}}\text{)}}$ is the predicted risk of clinical outcomes from the CHIME model for individual *i*. |
| $\mathbf{ln(} \frac{\boldsymbol{Observed}_{\boldsymbol{i}}}{\boldsymbol{Predicted}_{\boldsymbol{i}}}\mathbf{)=}\ln\left[ \mathbf{e}^{\mathbf{(}\boldsymbol{\gamma}_{\boldsymbol{CMS}}\mathbf{*}\boldsymbol{CMS}_{\boldsymbol{i}}\mathbf{+}\boldsymbol{\gamma}_{\boldsymbol{NCKUH}}\mathbf{*}\boldsymbol{NCKUH}_{\boldsymbol{i}}\mathbf{+}\boldsymbol{\gamma}_{\boldsymbol{NTUH}}\mathbf{*}\boldsymbol{NTUH}_{\boldsymbol{i}}\mathbf{-}\boldsymbol{\gamma}_{\boldsymbol{CMS}}\mathbf{)}} \right]$  $\mathbf{=}\boldsymbol{\gamma}_{\boldsymbol{NCKUH}}\mathbf{*}\boldsymbol{NCKUH}_{\boldsymbol{i}}\boldsymbol{+}\boldsymbol{\gamma}_{\boldsymbol{NTUH}}\mathbf{*}\boldsymbol{NTUH}_{\boldsymbol{i}}\boldsymbol{+}\boldsymbol{\gamma}_{\boldsymbol{CMS}}\mathbf{*}\left( \boldsymbol{CMS}\mathbf{-1} \right)$ (3) |
| Equation (3) is derived from Equation (2) under the assumption that the hazard ratios are constant over time. $\text{Observed}_{\text{i}}$ is the observed event rate of the outcome in cohort *i* (i.e., $\text{hazard}_{\text{it}}$ in Equation [2]) and $\text{Predicted}_{\text{i}}$ is the predicted value from the CHIME model (i.e., $\text{h}_{\text{t}}\text{*}\text{e}^{\text{(}\text{γ}_{\text{CMS}}\text{ }\text{+}\text{ }\text{β}_{\text{D.B.T.H }}\text{* }\text{D.B.T.H}_{\text{it}}\text{)}}$ in Equation [2]). Through Equation (3), we can measure the relative bias when applying the CHIME model in the NCKUH and NTUH populations that the present study aimed to estimate, which were $\text{e}^{\left( \text{ }\text{γ}_{\text{NCKUH}} \text{-}\text{ }\text{γ}_{\text{CMS}} \right)}$ and $\text{e}^{\left( \text{ }\text{γ}_{\text{NTUH}\text{ }} \text{-}{\text{ }\text{γ}}_{\text{CMS}} \right)}$, respectively. |
| $\mathbf{ln(} \frac{\boldsymbol{Observed}_{\boldsymbol{ij}}}{\boldsymbol{Predicted}_{\boldsymbol{ij}}}\mathbf{)=}\boldsymbol{\gamma}_{\boldsymbol{NCKUH}_{\boldsymbol{j}}}\mathbf{*}\boldsymbol{NCKUH}_{\boldsymbol{ij}}\boldsymbol{+}$ $\boldsymbol{\gamma}_{\boldsymbol{NTUH}_{\boldsymbol{j}}}\mathbf{*}\boldsymbol{NTUH}_{\boldsymbol{ij}}\boldsymbol{+}\boldsymbol{\gamma}_{\boldsymbol{CMS}}\mathbf{*}\left( \boldsymbol{CMS}\mathbf{-1} \right)$ (4) |
| If the recalibration was not satisfied after Equation (3) is applied, where differences between the observed and predicted risks were assumed to be homogeneous across the study patients within their healthcare systems, we further stratified the recalibration analyses by the risk stratum of predicted risks to determine whether the recalibration was improved. For example, $\text{γ}_{\text{NCKUH}_{\text{j}}}$ is the multipliers for subjects at different risk strata from NCKUH. The cut-off points of a risk stratum were determined based on the data distribution of risk differences across the deciles of predicted risks of clinical outcomes (as shown below).   |

Reference: the recalibration method was modified based on Shao et al.’s study (Value Health. 2019;22(12):1402-1409.).

Supplementary Table 5. Demographic and clinical characteristics of patients with type 2 diabetes from NCKUH

|  | Overall cohort | Training dataset (MI/HF) | Testing dataset (MI/HF) | SMD* | Training dataset (stroke^†^) | Testing dataset (stroke^†^) | SMD* |
| --- | --- | --- | --- | --- | --- | --- | --- |
| Sample size | 11,740 | 5,870 | 5,870 |  | 5,871 | 5,869 |  |
| Follow-up period (mean, SD) | 4.5 (0.9) | 4.5 (0.9) | 4.5 (0.9) |  | 4.5 (0.9) | 4.5 (0.9) |  |
| Demographic characteristics |  |  |  |  |  |  |  |
| Age, years (mean, SD) | 63.3 (12.0) | 63.4 (12.1) | 63.3 (11.9) | 0.009 | 63.2 (12.0) | 63.4 (12.0) | -0.015 |
| Female (%) | 45.7% | 45.6% | 45.8% | -0.006 | 45.4% | 45.8% | -0.013 |
| Smoking status (%) |  |  |  |  |  |  |  |
| Current | 6.3% | 6.7% | 5.9% | 0.034 | 6.6% | 6.1% | 0.021 |
| Quit | 3.1% | 3.5% | 2.7% | 0.044 | 3.0% | 3.1% | -0.003 |
| Never | 90.6% | 89.8% | 91.4% | -0.054 | 90.4% | 90.8% | -0.016 |
| Medical history (%) |  |  |  |  |  |  |  |
| Atrial fibrillation | 2.9% | 3.0% | 2.7% | 0.024 | 2.9% | 2.8% | 0.001 |
| Stroke | 9.1% | 8.7% | 9.4% | -0.025 | 9.1% | 9.0% | 0.000 |
| Ischemic heart disease | 20.0% | 19.6% | 20.5% | -0.023 | 20.1% | 19.9% | 0.006 |
| Myocardial infarction | 3.0% | 3.0% | 3.1% | -0.006 | 3.0% | 3.0% | 0.000 |
| Heart failure | 5.4% | 5.5% | 5.4% | 0.002 | 5.0% | 5.9% | -0.041 |
| Peripheral vascular disease | 1.9% | 1.9% | 2.0% | -0.004 | 1.9% | 2.0% | -0.009 |
| Amputation | 0.1% | 0.1% | 0.1% | 0.010 | 0.1% | 0.1% | 0.000 |
| Ulcer | 0.2% | 0.2% | 0.2% | -0.004 | 0.1% | 0.2% | -0.028 |
| Medication use (%) |  |  |  |  |  |  |  |
| Insulin | 14.8% | 14.8% | 14.8% | -0.002 | 14.6% | 15.0% | -0.014 |
| Glucose-lowering agents (except insulin) | 87.9% | 88.1% | 87.7% | 0.014 | 87.7% | 88.2% | -0.016 |
| Anti-hypertensive drugs | 72.3% | 72.6% | 72.0% | 0.013 | 72.0% | 72.6% | -0.013 |
| Anti-hyperlipidemia drugs | 63.5% | 63.6% | 63.3% | 0.005 | 63.3% | 63.6% | -0.006 |
| Anti-thrombotic therapy | 37.6% | 37.4% | 37.7% | -0.007 | 37.6% | 37.6% | 0.000 |
| Physical examination (mean, SD) |  |  |  |  |  |  |  |
| BMI, kg/m^2^ | 26.2 (3.7) | 26.2 (3.7) | 26.2 (3.7) | -0.011 | 26.2 (3.6) | 26.3 (3.8) | -0.019 |
| SBP, mmHg | 131.4 (9.3) | 131.4 (9.2) | 131.5 (9.3) | -0.010 | 131.5 (9.3) | 131.4 (9.3) | 0.013 |
| DBP, mmHg | 78.2 (5.8) | 78.2 (5.7) | 78.3 (5.8) | -0.002 | 78.3 (5.8) | 78.2 (5.7) | 0.014 |
| Laboratory data (mean, SD) |  |  |  |  |  |  |  |
| HbA1c, % | 7.7 (1.6) | 7.7 (1.7) | 7.7 (1.6) | -0.013 | 7.7 (1.6) | 7.7 (1.6) | 0.000 |
| TC, mg/dL | 170.5 (36.0) | 170.2 (35.0) | 170.9 (36.8) | -0.022 | 170.6 (34.9) | 170.4 (37.0) | 0.007 |
| TG, mg/dL | 154.4 (119.1) | 153.0 (110.8) | 155.7 (126.9) | -0.023 | 154.2 (119.3) | 154.5 (119.0) | -0.002 |
| HDL, mg/dL | 48.7 (13.5) | 48.9 (13.6) | 48.5 (13.4) | 0.026 | 48.7 (13.3) | 48.7 (13.6) | -0.002 |
| LDL, mg/dL | 106.0 (29.9) | 105.7 (29.9) | 106.4 (29.9) | -0.021 | 106.3 (29.8) | 105.7 (30.0) | 0.020 |
| Scr, mg/dL | 1.0 (1.0) | 1.0 (1.0) | 1.0 (1.0) | 0.005 | 1.0 (0.9) | 1.0 (1.1) | -0.040 |
| eGFR^‡^, ml/min/1.73m^2^ | 87.4 (25.9) | 87.1 (26.2) | 87.6 (25.7) | -0.017 | 88.0 (25.5) | 86.7 (26.3) | 0.048 |
| UACR, mg/g | 201.3 (472.6) | 199.1 (466.5) | 203.5 (478.6) | -0.009 | 190.1 (450.4) | 212.5 (493.5) | -0.048 |
| WBC, 10^9^/L | 9.4 (2.3) | 9.4 (2.2) | 9.4 (2.4) | 0.001 | 9.4 (2.2) | 9.4 (2.4) | -0.021 |
| Hgb, g/dL | 12.7 (1.6) | 12.7 (1.6) | 12.7 (1.6) | 0.003 | 12.7 (1.6) | 12.7 (1.6) | 0.031 |

Abbreviations: NCKUH, National Cheng Kung University Hospital; MI, myocardial infarction; HF, heart failure; SMD, standardized mean difference; SD, standard deviation; BMI, body mass index; SBP, systolic blood pressure; DBP, diastolic blood pressure; TC, total cholesterol; TG, triglycerides; HDL, high-density lipoprotein; LDL, low-density lipoprotein; Scr, serum creatinine; eGFR, estimated glomerular filtration rate; UACR, urine albumin-creatinine ratio; WBC, white blood cell; Hgb, hemoglobin.

Notes:

*An absolute value of SMD > 0.1 represents a statistically significant difference in patient characteristics between training and testing datasets.

^†^An imbalance was observed in the stroke event risks between training (6.41%) and testing (7.19%) datasets after the random split of the overall cohort. A stratified randomization procedure (strata: the history of stroke and atrial fibrillation) was further applied to generate the training and testing datasets for the stroke outcome.

^‡^eGFR was estimated based on the serum creatinine level using the Chronic Kidney Disease Epidemiology Collaboration (CKD-EPI) equation.

Supplementary Table 6. Demographic and clinical characteristics of patients with type 2 diabetes from NTUH

|  | Overall cohort | Training dataset | Testing dataset | SMD* |
| --- | --- | --- | --- | --- |
| Sample size | 20,313 | 10,157 | 10,156 |  |
| Follow-up period (mean, SD) | 4.3 (0.9) | 4.3 (0.9) | 4.3 (0.9) |  |
| Demographic characteristics |  |  |  |  |
| Age (mean, SD) | 64.8 (11.6) | 64.8 (11.6) | 64.8 (11.6) | 0.001 |
| Female (%) | 47.1% | 47.2% | 46.9% | 0.006 |
| Smoking status^†^ (%) |  |  |  |  |
| Current | 0% | 0% | 0% | 0 |
| Quit | 0% | 0% | 0% | 0 |
| Never | 100% | 100% | 100% | 0 |
| Medical history (%) |  |  |  |  |
| Atrial fibrillation | 3.1% | 3.1% | 3.1% | -0.002 |
| Stroke | 3.7% | 3.6% | 3.7% | -0.001 |
| Ischemic heart disease | 24.1% | 24.3% | 23.8% | 0.013 |
| Myocardial infarction | 2.4% | 2.4% | 2.3% | 0.008 |
| Heart failure | 3.4% | 3.5% | 3.4% | 0.006 |
| Peripheral vascular disease | 3.8% | 3.8% | 3.8% | -0.001 |
| Amputation | 0.0% | 0.0% | 0.0% | 0.010 |
| Ulcer | 0.1% | 0.2% | 0.1% | 0.022 |
| Medication use (%) |  |  |  |  |
| Insulin | 14.9% | 14.8% | 14.9% | -0.002 |
| Glucose-lowering agents (except insulin) | 82.0% | 82.3% | 81.7% | 0.016 |
| Anti-hypertensive drugs | 71.8% | 72.2% | 71.5% | 0.015 |
| Anti-hyperlipidemia drugs | 26.3% | 26.2% | 26.3% | -0.003 |
| Anti-thrombotic therapy | 29.2% | 29.2% | 29.1% | 0.001 |
| Physical examination (mean, SD) |  |  |  |  |
| BMI, kg/m^2^ | 25.9 (3.4) | 25.9 (3.4) | 25.9 (3.4) | -0.001 |
| SBP, mmHg | 131.8 (9.4) | 131.7 (9.3) | 131.8 (9.4) | -0.012 |
| DBP, mmHg | 74.4 (6.4) | 74.4 (6.4) | 74.5 (6.4) | -0.016 |
| Laboratory data (mean, SD) |  |  |  |  |
| HbA1c, % | 7.2 (1.4) | 7.2 (1.4) | 7.2 (1.4) | 0.012 |
| TC, mg/dL | 176.1 (34.0) | 176.2 (34.8) | 175.9 (33.2) | 0.010 |
| TG, mg/dL | 161.7 (122.9) | 162.7 (127.5) | 160.8 (118.1) | 0.015 |
| HDL, mg/dL | 45.0 (10.1) | 45.0 (10.2) | 45.0 (10.0) | 0.000 |
| LDL, mg/dL | 104.4 (24.9) | 104.3 (25.0) | 104.4 (24.8) | -0.005 |
| Scr, mg/dL | 1.1 (1.0) | 1.1 (1.0) | 1.1 (0.9) | 0.026 |
| eGFR^‡^, ml/min/1.73m^2^ | 80.6 (23.6) | 80.3 (23.6) | 80.8 (23.5) | -0.021 |
| UACR, mg/g | 118.8 (111.7) | 119.4 (110.0) | 118.3 (113.5) | 0.010 |
| WBC, 10^9^/L | 7.4 (1.9) | 7.4 (1.9) | 7.4 (1.9) | 0.022 |
| Hgb^b^, g/dL | 12.5 (0) | 12.5 (0) | 12.5 (0) | Not applicable |

Abbreviations: NTUH, National Taiwan University Hospital; SMD, standardized mean difference; SD, standard deviation; BMI, body mass index; SBP, systolic blood pressure; DBP, diastolic blood pressure; TC, total cholesterol; TG, triglycerides; HDL, high-density lipoprotein; LDL, low-density lipoprotein; Scr, serum creatinine; eGFR, estimated glomerular filtration rate; UACR, urine albumin-creatinine ratio; WBC, white blood cell; Hgb, hemoglobin.

Notes:

*An absolute value of SMD > 0.1 represents a statistically significant difference in patient characteristics between training and testing datasets.

^†^Smoking and hemoglobin data were not available in the NTUH database. We assumed that all study patients from NTUH were non-smokers and had a hemoglobin level of 12.5 g/dL.

^‡^eGFR was estimated based on the serum creatinine level using the Chronic Kidney Disease Epidemiology Collaboration (CKD-EPI) equation.

Supplementary Table 7. Observed cardiovascular outcomes among patients with type 2 diabetes identified from electronic health records of NCKUH and NTUH

|  | Number of events | Follow-up (PYs) | Event rate (/100 PYs) | Event risk (%) |
| --- | --- | --- | --- | --- |
| NCKUH | | | | |
| Overall cohort | | | | |
| Myocardial infarction | 279 | 52,032 | 0.536 | 2.38% |
| Stroke | 798 | 50,836 | 1.570 | 6.80% |
| Heart failure | 1,015 | 50,214 | 2.021 | 8.65% |
| Training dataset |  |  |  |  |
| Myocardial infarction | 143 | 25,982 | 0.550 | 2.44% |
| Stroke | 392 | 25,396 | 1.544 | 6.68% |
| Heart failure | 508 | 25,081 | 2.025 | 8.65% |
| Testing dataset |  |  |  |  |
| Myocardial infarction | 136 | 26,050 | 0.522 | 2.32% |
| Stroke | 406 | 25,440 | 1.596 | 6.92% |
| Heart failure | 507 | 25,133 | 2.017 | 8.64% |
| NTUH | | | | |
| Overall cohort | | | | |
| Myocardial infarction | 309 | 86,969 | 0.355 | 1.52% |
| Stroke | 849 | 85,544 | 0.992 | 4.18% |
| Heart failure | 720 | 86,122 | 0.836 | 3.54% |
| Training dataset |  |  |  |  |
| Myocardial infarction | 153 | 43,432 | 0.352 | 1.51% |
| Stroke | 434 | 42,710 | 1.016 | 4.27% |
| Heart failure | 362 | 42,989 | 0.842 | 3.56% |
| Testing dataset |  |  |  |  |
| Myocardial infarction | 156 | 43,415 | 0.359 | 1.54% |
| Stroke | 414 | 42,714 | 0.969 | 4.08% |
| Heart failure | 358 | 43,011 | 0.832 | 3.53% |

Abbreviations: NCKUH, National Cheng Kung University Hospital; NTUH, National Taiwan University Hospital; PYs, person-years.

Supplementary Table 8. Patient characteristics of development cohorts for UKPDS-OM2, RECODe, and CHIME models and study populations in present study

|  | UKPDS-OM2 | RECODe | CHIME model | NCKUH | NTUH |
| --- | --- | --- | --- | --- | --- |
| Sample size | 5,102 | 9,635 | 97,628 | 11,740 | 20,313 |
| Study setting | Clinical trial | Clinical trial | Real-world practice | Real-world practice | Real-world practice |
| Baseline characteristics |  |  |  |  |  |
| Demographic characteristics |  |  |  |  |  |
| Age (mean, SD) | 53.3 (8.6) | 62.8 (6.7) | 60.0 (12.6) | 63.3 (12.0) | 64.8 (11.6) |
| Female (%) | 39% | 38% | 43.5% | 45.7% | 47.1% |
| Smoking status (%) |  |  |  |  |  |
| Current | 31% | 12% | 13.7% | 6.3% | 0% |
| Quit | 35% | NR | 18.8% | 3.1% | 0% |
| Never | 34% | NR | 67.5% | 90.6% | 100% |
| Medical history (%) |  |  |  |  |  |
| Atrial fibrillation | NR | NR* | 1.7% | 2.9% | 3.1% |
| Stroke | NR | NR* | 4.0% | 9.1% | 3.7% |
| Ischemic heart disease | 0% | NR* | 2.8% | 20.0% | 24.1% |
| Myocardial infarction | 0% | NR* | 1.5% | 3.0% | 2.4% |
| Heart failure | 0% | NR* | 1.6% | 5.4% | 3.4% |
| Peripheral vascular disease | NR | NR* | 0.4% | 1.9% | 3.8% |
| Amputation | NR | NR* | 0.2% | 0.1% | 0.0% |
| Ulcer | NR | NR* | 0.4% | 0.2% | 0.1% |
| Medication use (%) |  |  |  |  |  |
| Insulin | NR | 35% | 3.6% | 14.8% | 14.9% |
| Glucose-lowering agents (except insulin) | NR | 83% | 18.2% | 87.9% | 82.0% |
| Anti-hypertensive drugs | 12% | 84% | 35.2% | 72.3% | 71.8% |
| Anti-hyperlipidemia drugs | 0.8% | 64% | 7.6% | 63.5% | 26.3% |
| Anti-thrombotic therapy | NA | 3% | NA | 37.6% | 29.2% |
| Physical examination (mean, SD) |  |  |  |  |  |
| BMI, kg/m^2^ | 27.5 (5.2) | 32.2 (5.4) | 25.6 (4.3) | 26.2 (3.7) | 25.9 (3.4) |
| SBP, mmHg | 135 (20) | 136.5 (17.1) | 135.3 (15.4) | 131.4 (9.3) | 131.8 (9.4) |
| DBP, mmHg | 82 (10) | 74.9 (10.7) | 77.6 (9.7) | 78.2 (5.8) | 74.4 (6.4) |
| Laboratory data (mean, SD) |  |  |  |  |  |
| HbA1c, % | 7.1 (1.5) | 8.3 (1.1) | 7.8 (1.7) | 7.7 (1.6) | 7.2 (1.4) |
| TC, mg/dL | 209.0 (42.6) | 183.2 (41.7) | NR | 170.5 (36.0) | 176.1 (34.0) |
| TG, mg/dL | 208.1 (NA) | 190.7 (145.8) | 141.7 (79.7) | 154.4 (119.1) | 161.7 (122.9) |
| HDL, mg/dL | 41.4 (9.3) | 41.8 (11.6) | 50.3 (11.6) | 48.7 (13.5) | 45.0 (10.1) |
| LDL, mg/dL | 135.5 (38.7) | 104.7 (33.8) | 116.0 (30.9) | 106.0 (29.9) | 104.4 (24.9) |
| Scr, mg/dL | 0.9 (NA) | 0.9 (0.2) | NR | 1.0 (1.0) | 1.1 (1.0) |
| eGFR, ml/min/1.73m^2^ | NR | 90.9 (27.3) | 92.2 (28.4) | 87.4 (25.9) | 80.6 (23.6) |
| UACR, mg/g | NR | 99.2 (359.4) | NR | 201.3 (472.6) | 118.8 (111.7) |
| WBC, 10^9^/L | NR | NR | 8.0 (2.3) | 9.4 (2.3) | 7.4 (1.9) |
| Hgb, g/dL | NR | NR | 13.7 (1.7) | 12.7 (1.6) | 12.5 (0) |
| Event risks of cardiovascular outcomes |  |  |  |  |  |
| Myocardial infarction | 19.87% | 9.13% | 5.34% | 2.38% | 1.52% |
| Stroke | 9.88% | 2.04% | 10.27% | 6.80% | 4.18% |
| Heart failure | 6.88% | 4.71% | 7.62% | 8.65% | 3.54% |

Abbreviations: UKPDS-OM2, UK Prospective Diabetes Study Outcomes Model 2; RECODe, Risk Equations for Complications Of type 2 Diabetes; CHIME, Chinese Hong Kong Integrated Modeling and Evaluation; NCKUH, National Cheng Kung University Hospital; NTUH, National Taiwan University Hospital; SD, standard deviation; NR, not reported; BMI, body mass index; SBP, systolic blood pressure; DBP, diastolic blood pressure; TC, total cholesterol; TG, triglycerides; HDL, high-density lipoprotein; LDL, low-density lipoprotein; Scr, serum creatinine; eGFR, estimated glomerular filtration rate; UACR, urine albumin-to-creatinine ratio; WBC, white blood cell; Hgb, hemoglobin.

*The RECODe study reported patient’s history of the composite cardiovascular disease (36%) instead of individual cardiovascular diseases.

Supplementary Figure 1. Identification of study populations, risk predictors, and cardiovascular outcomes from NCKUH and NTUH EHRs

Abbreviations: NCKUH, National Cheng Kung University; NTUH, National Taiwan University Hospital; EHR, electronic health record; T2D, type 2 diabetes.

Notes: Patients with at least two type 2 diabetes diagnoses during 2015-2018 were identified from the NCKUH EHRs and followed until death or December 31, 2019, whichever came first. In the NTUH EHRs, patients with at least two type 2 diabetes diagnoses during 2013-2016 were identified and followed until death or December 31, 2017, whichever came first.

Supplementary Figure 2. Study flow of risk equation adaptations for target study populations

Abbreviations: AUROC, area under the receiver operating characteristics curve; GND, Greenwood-Nam-D’Agostino.

Supplementary Figure 3. Study flow diagram in (a) National Cheng Kung University Hospital and (b) National Taiwan University Hospital


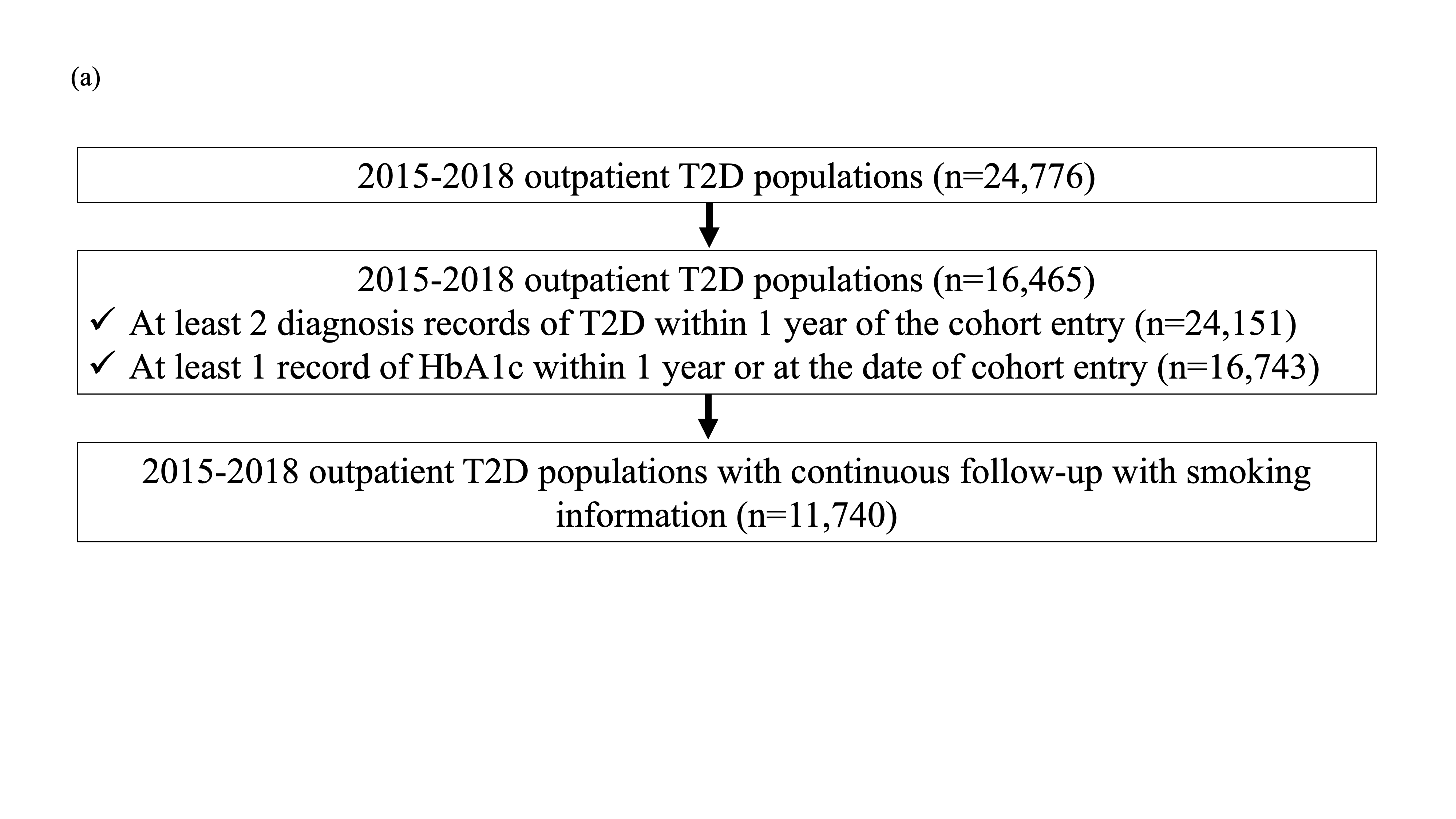


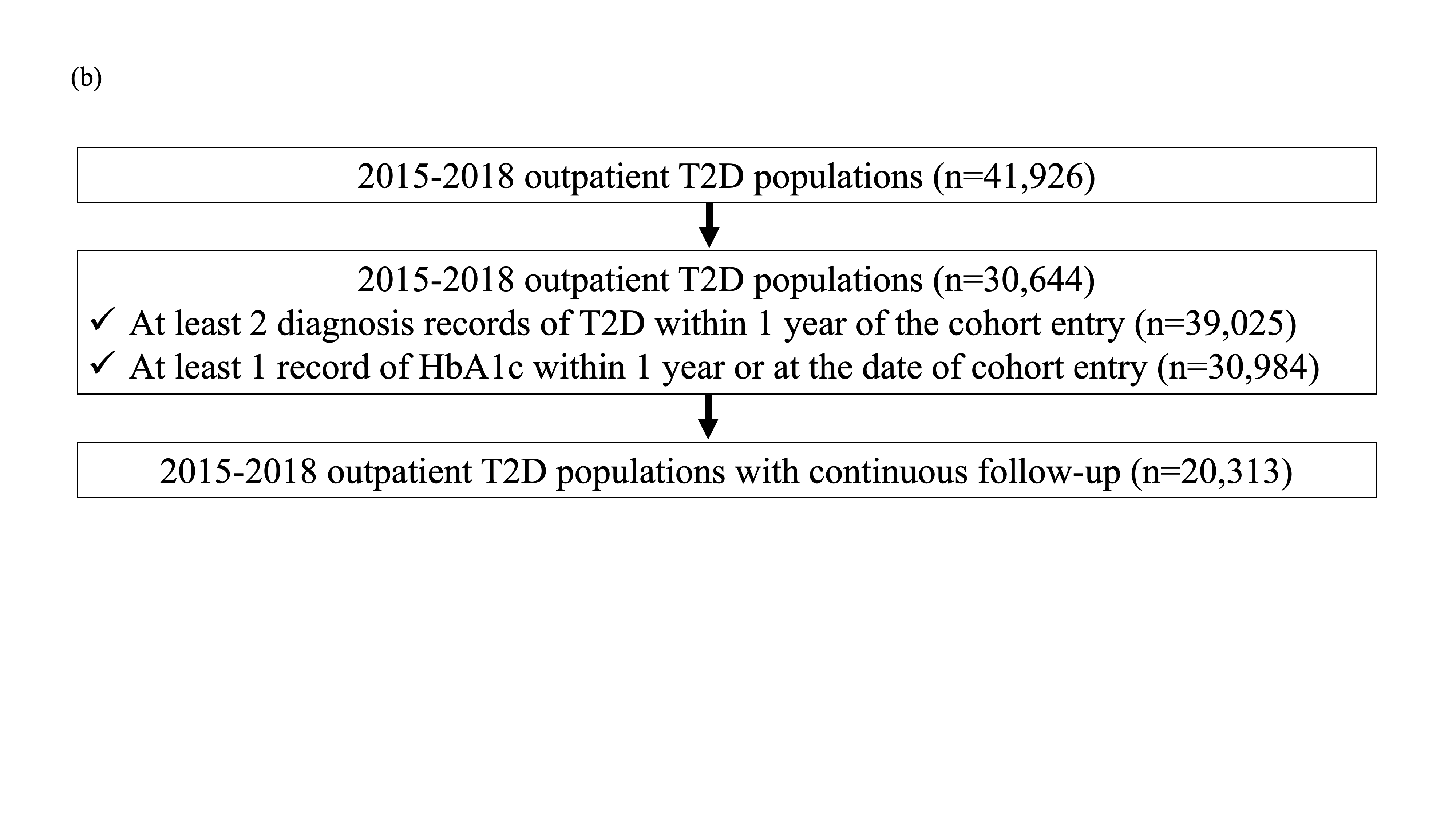


Abbreviation: T2D, type 2 diabetes.

Note: The smoking information was not available in National Taiwan University Hospital.

Supplementary Figure 4. Calibrations of MI risk equations from UKPDS-OM2, RECODe, and CHIME models for patients with type 2 diabetes from (a) NCKUH and (b) NTUH

Abbreviations: MI, myocardial infarction; UKPDS-OM2, UK Prospective Diabetes Study Outcomes Model 2; RECODe, Risk Equations for Complications Of type 2 Diabetes; CHIME, Chinese Hong Kong Integrated Modeling and Evaluation; T2D, type 2 diabetes; NCKUH, National Cheng Kung University Hospital; NTUH, National Taiwan University Hospital.

Supplementary Figure 5. Calibrations of stroke risk equations from UKPDS-OM2, RECODe, and CHIME models for patients with type 2 diabetes from (a) NCKUH and (b) NTUH

Abbreviations: UKPDS-OM2, UK Prospective Diabetes Study Outcomes Model 2; RECODe, Risk Equations for Complications Of type 2 Diabetes; CHIME, Chinese Hong Kong Integrated Modeling and Evaluation; T2D, type 2 diabetes; NCKUH, National Cheng Kung University Hospital; NTUH, National Taiwan University Hospital.

Supplementary Figure 6. Calibrations of HF risk equations from UKPDS-OM2, RECODe, and CHIME models for patients with type 2 diabetes from (a) NCKUH and (b) NTUH

Abbreviations: HF, heart failure; UKPDS-OM2, UK Prospective Diabetes Study Outcomes Model 2; RECODe, Risk Equations for Complications Of type 2 Diabetes; CHIME, Chinese Hong Kong Integrated Modeling and Evaluation; T2D, type 2 diabetes; NCKUH, National Cheng Kung University Hospital; NTUH, National Taiwan University Hospital.
